# Gr1^int/high^ Cells Dominate the Early Phagocyte Response to Mycobacterial Lung Infection in Mice

**DOI:** 10.3389/fmicb.2019.00402

**Published:** 2019-03-08

**Authors:** Brin M. Ryder, Sarah K. Sandford, Kate M. Manners, James P. Dalton, Siouxsie Wiles, Joanna R. Kirman

**Affiliations:** ^1^Department of Microbiology and Immunology, University of Otago, Dunedin, New Zealand; ^2^Bioluminescent Superbugs Lab, Department of Molecular Medicine and Pathology, University of Auckland, Auckland, New Zealand

**Keywords:** lung, phagocytes, BCG, tuberculosis, neutrophils, dendritic cells, monocytes, macrophages

## Abstract

Lung infection by *Mycobacterium tuberculosis* is characterized by chronic infection of lung-resident macrophages, long considered to be the primary hosts and determinants of the outcome of the early immune response. Although alveolar macrophages are well-known to host intracellular mycobacteria at later stages of disease, little is known about the earliest events of the innate immune response. The phagocytes that take up mycobacteria immediately following infection, and how the early lung phagocyte response is altered by vaccination with *M. bovis* bacille Calmette-Guérin (BCG) were unknown. Using BCG expressing the bright red fluorescent protein tdTomato and flow cytometry, we modeled early infection in C57BL/6 mice and tracked phagocyte population kinetics and uptake of mycobacteria, to better understand the involvement of specific phagocyte subsets. By 1 day post-infection, dose-dependent accumulation of neutrophils was observed and surprisingly, granulocytes comprised a greater proportion of infected phagocytes than alveolar macrophages. By 7 days post-infection alveolar macrophages had become the dominant BCG-associated phagocytes. Prior mucosal BCG exposure provided immunized mice with greater frequencies and numbers of lung macrophage subsets, and a significantly greater proportion of alveolar macrophages expressed CD11b prior to and following challenge infection. These data provide the first evidence of granulocytes as the dominant infected phagocyte subset early after mycobacterial infection, and highlight enhanced recruitment of lung macrophages as a factor associated with protection in BCG-immunized mice.

## Introduction

Exposure to aerosolized *Mycobacterium tuberculosis* (Mtb) can lead to development of a chronic lung infection, characterized by persistent intracellular infection of alveolar macrophages (AMΦ) (Cunningham et al., 1925; Scriba et al., 2017). Due to the chronic nature of tuberculosis (TB) infection, studies of responding phagocytic cells have focused on established, rather than early, phases of infection (Srivastava et al., 2014). Examination of infected animal lungs and bronchoalveolar lavage (BAL) fluid of human patients has revealed that AMΦ are primary hosts to intracellular mycobacteria during chronic infection (Cunningham et al., 1925; Scriba et al., 2017). During early infection, it is presumed that AMΦ are the primary bacterial hosts (Yoshikai, 2006; Orme et al., 2015), though direct evidence is limited.

Older studies, using light microscopy with stained tissue sections, suggest the involvement of other phagocytic cells, including neutrophils, during the early stages of mycobacterial infection (Pottenger, 1948). Electron microscopy studies have shown that AMΦ take up mycobacteria immediately after intranasal (i.n.) mycobacterial infection, and within 4 h, some mycobacteria are transcytosed across respiratory mucosa and shuttled to local lymph nodes by migratory peripheral blood-derived phagocytes (Teitelbaum et al., 1999). It has also been shown that dendritic cells can be recruited to the lung within hours of i.n. mycobacterial infection, and associate with the invading mycobacteria (Lagranderie et al., 2003; Reljic et al., 2005). Although these older studies were unable to include sufficient antibodies against cell surface markers to clearly define the relative contribution of monocytes, dendritic cells and macrophages, they demonstrated that phagocytes other than AMΦ could take up the bacilli within hours of exposure.

It is possible that the early phagocytic response in the lung following Mtb infection might dictate whether there is early control or progressive disease (van Crevel et al., 2002). Moreover, with the recent discovery of resident memory T cells, there is great interest in pursuing mucosal routes of immunization using live mycobacterial vaccines; yet the contribution of phagocytes to respiratory mucosal vaccination is unknown. The discovery that innate cells, such as monocytes, can acquire a “trained” phenotype following exposure to BCG, suggests phagocytic responses to immunization may be critical for establishing immune protection (Kirman et al., 2016). The paucity of evidence for the prevailing dogma that AMΦ dominate during early mycobacterial infection (Srivastava et al., 2014) led us to ask which phagocytes contribute to the early response to a primary mycobacterial exposure as well as early during a recall response.

In the steady-state, myeloid phagocytes can be segregated into distinct populations based on their developmental and spatial origins, and effector function. In the lungs of mice, alveolar macrophages, interstitial macrophages, dendritic cells and monocytes can be easily distinguished by multi-parameter flow cytometry (Gibbings and Jakubzick, 2018). Under inflammatory conditions, such as during Mtb infection or BCG immunization, it becomes more challenging to distinguish discrete subsets as new phagocytes, including neutrophils and monocytes, are recruited to the lungs and alter expression of cell surface receptors as they become differentiated (Srivastava et al., 2014; Norris and Ernst, 2018).

Neutrophils (Ly6G+/Gr1^high^) infiltrate the lungs of mice from the first day of Mtb infection, however, their role at this early stage is unclear (Barrios-Payán et al., 2006). At 2 weeks after infection with Mtb-mCherry, neutrophils represented the majority of infected phagocytes (Huang et al., 2018). Whether early neutrophil infiltration to the lungs is related to the infective dose is unclear, as is the degree to which early infiltrating neutrophils interact with mycobacteria. Moreover, whether the neutrophil response is altered following mucosal vaccination is unknown.

In C57BL/6 (B6) mice, circulating Ly6C^high^/Gr1+ (CCR2+; classical) and CX3CR1^high^ (non-classical) monocytes broadly reflect the CD14+ and CD16+ monocyte subsets found in humans (Serbina et al., 2008). Ly6C^high^/Gr1+ monocytes are preferentially recruited to acutely inflamed tissue (Hume, 2006; Srivastava et al., 2014) where they rapidly differentiate, downregulate Ly6C and upregulate surface expression of CX3CR1 (Serbina et al., 2008; Norris and Ernst, 2018). At 4 weeks post-infection with Mtb in B6 mice, adoptively transferred Ly6C^high^ monocytes are recruited to the lungs within 40 h, and rapidly differentiate into dendritic cell and macrophage subsets with a high rate of turnover (Norris and Ernst, 2018). The anti-mycobacterial efficacy of lung-infiltrating monocytes 4 weeks after infection with Mtb is modulated by the lung inflammatory milieu, demonstrated by the impairment of bacterial restriction in mice treated with i.n. poly-IC (Antonelli et al., 2010). Restriction of mycobacteria by lung phagocytes is dependent on IL-1α/β in mice (Mayer-Barber et al., 2010), release of which is inhibited by type I IFN signaling induced in both mouse (Mayer-Barber et al., 2011) and human macrophages (Novikov et al., 2011) infected by virulent mycobacteria. The dynamics of phagocyte subsets and contribution of infiltrating monocytes to uptake of mycobacteria during earlier phases of infection remain to be established.

In this study, we exposed B6 mice to fluorescent *Mycobacterium bovis* BCG and used multi-parametric flow cytometry to characterize the highly dynamic early phagocytic response in the lung. During the first day of infection there was dose-dependent infiltration of Gr1^int^ and Gr1^high^ granulocytes into the lungs, which were the dominant infected phagocyte populations. Macrophages then predominated by 7 days post-infection (p.i.). In BCG-immunized mice, macrophage subsets were present in greater number prior to infection and more were recruited after infection, compared to unimmunized mice. These data reveal a previously unappreciated role for neutrophils as the primary mycobacteria-associated phagocyte in the lung, at the earliest stages of infection.

## Materials and Methods

### Bacterial Culture

*Mycobacterium bovis* BCG Japan was obtained from the ATCC (#35737) and grown at 37°C in Middlebrook 7H9 broth (Fort Richard Laboratories, NZ), supplemented with 10% Middlebrook OADC enrichment media (Fort Richard Laboratories), 0.5% glycerol (Sigma-Aldrich, St. Louis, MO, United States) and 0.05% Tween-80 (Sigma-Aldrich). *M. bovis* BCG Pasteur 1173P2 was cultured, similarly.

To express the fluorescent protein tdTomato in BCG Japan, the cells were transformed using pTEC27 (Addgene plasmid #30182, RRID:Addgene_30182) (Takaki et al., 2013) as previously described (Parish and Stoker, 1998). BCG tdTomato was cultured in Dubos Tween Albumin broth (Becton, Dickinson and Company, Sparks, MD, United States) with 10% Middlebrook OADC enrichment media and 50 μg/mL hygromycin B (HygB; Sigma-Aldrich) to select for plasmid carriage. BCG were harvested at mid-log phase (OD_600_ ≈ 0.4), washed twice in sterile Dulbecco’s phosphate-buffered saline (PBS; Invitrogen, NZ) with 0.05% Tween-80 (Tween-PBS), and frozen at -80°C. Defrosted BCG cryotubes were briefly sonicated then diluted in Tween-PBS.

BCG were enumerated by spot-plating on 7H11 agar containing 10% OADC, 50 μg/mL Carbenicillin (Life Technologies, Grand Island, NY, United States), 24 μg/mL Polymyxin B (Life Technologies), with or without the addition of 50 μg/mL HygB. Lung tissue was homogenized (T18 Basic S5; IKA Works, NC, United States) in Tween-PBS for serial dilution and spot-plating. Plates were cultured at 37°C for 2–3 weeks, before colonies were counted to determine numbers of colony forming units (CFU).

### Animals

C57BL/6J mice (RRID:IMSR_JAX:000664) were obtained from Jackson Laboratories, bred and housed at the University of Otago in individually-ventilated cages under specific pathogen-free conditions (Supplementary Table 4). Age-matched, male mice between 6 and 9 weeks old were used.

To facilitate i.n. infection with BCG tdTomato and immunization with BCG Pasteur, mice were anaesthetized by intraperitoneal (i.p.) injection of ketamine (87 mg/Kg; Phoenix Pharm Distributors Ltd., New Zealand) and xylazine (2.6 mg/Kg; Phoenix Pharm Distributors Ltd.). Indicated doses of BCG tdTomato or 10^5^ CFU of BCG Pasteur were pipetted dropwise onto the external nares until inhaled. Mice were euthanized for tissue harvest by i.p. injection of sodium pentobarbitone (150 mg/Kg; Provet NZ Pty Limited, Auckland, New Zealand).

All experiments were conducted in accordance with the Animal Welfare Act (1999) and with approval from the University of Otago Animal Ethics Committee.

### Lung Harvest and Single Cell Suspension

Lungs were perfused prior to removal, by injection of PBS into the right ventricle of the heart. To obtain single-cell suspensions, lungs were removed, chopped into small pieces, and incubated in incomplete Iscove’s Modified Dulbecco’s Medium (IMDM) containing 2.4 mg/mL Collagenase I (Life Technologies) and 0.12 mg/mL DNase I (Roche Diagnostics GmbH, Mannheim, Germany) for 1 h at 37°C with 5% CO_2_. Tissue chunks were gently mashed through 70 μm nylon strainers. Cells were counted using Trypan Blue exclusion in a haemocytometer.

### Flow Cytometry

Antibody labeling was performed shielded from light at 4°C. Before staining, F_C_γ receptors II/III were blocked with anti-CD16/32 antibody (clone 2.4G2; produced in-house) in FACS buffer (PBS with 0.5% FCS and 1 mM EDTA). Live/dead staining was carried out prior to labeling with cell surface antibodies. Cells were fixed in neutral-buffered Formalin (4% formaldehyde; Sigma-Aldrich). For subtyping Gr1+ cells, anti-Ly6C and anti-Ly6G were diluted and incubated in Brilliant Stain Buffer (BD Pharmingen) prior to fixation and applying anti-Gr1 antibody suspended in FACS buffer in a second surface-staining step.

Data were acquired using a BD LSR Fortessa with violet (405 nm), blue (488 nm), yellow-green (561 nm) and red (640 nm) lasers. The antibody panel and optical configuration are provided in Supplementary Tables 1, 2. Data were acquired using FACS Diva and analyzed using FlowJo 10.5.0 (Tree Star, Ashland, OR, United States). Gates were set against unstained and fluorescence-minus-one controls. At least 10^6^ events were recorded per sample.

### Cell-Sorting and Confocal Microscopy

Lung cells were harvested, processed to single cell suspension and stained for cytometry as described in Supplementary Table 1. Cells were sorted using a BD FACSAria IIu with violet (405 nm), blue (488 nm) and red (633 nm) lasers. Singlet, live, large (FSC^int/high^) or granular (SSC^int/high^) tdTomato+ events were sorted onto slides for microscopy on an Olympus FV1200 confocal microscope with yellow-green (543 nm) and red (633 nm) lasers. Confocal images were processed in Fiji/ImageJ 1.51n.

### Statistical Analyses

Significance of effects due to infection (fold-differences for each infected sample over median values of uninfected groups) were tested against the null hypothesis (μ = 1.0) using a one-sample signed Wilcoxon rank test. As normality and equality of variance were not consistent in all groups, non-parametric tests were used for effects of dose and time. Multifactorial data were unrolled to perform Kruskal-Wallis one-way analysis of variance, adjusted for multiplicity with Dunn’s post-test, per factor (dose and time; 3 Kruskal-Wallis tests each). Pairwise comparisons were made using two-tailed Mann–Whitney *U*-test. Analyses were conducted in RStudio 1.0.136 (RStudio, Inc., Boston, MA, United States) and R 3.3.1 (R Foundation for Statistical Computing, Vienna, Austria) for Wilcoxon rank tests or Graphpad Prism 7.04 (Graphpad Software Inc., San Diego, CA, United States) for Kruskal–Wallis and Mann–Whitney tests.

## Results

### Fluorescent Mycobacteria Are Detectable in Mouse Lung Phagocytes During Early Infection

**FIGURE 1:**
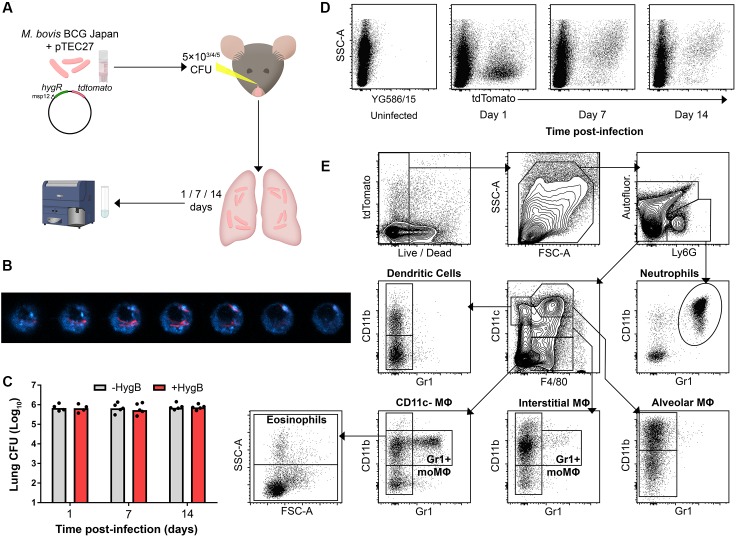
Fluorescent mycobacteria are detectable in mouse lung phagocytes during early infection. **(A)** Model for early mycobacterial lung infection. Hygromycin B (HygB) selects for tdTomato-expressing BCG in culture. BCG tdTomato was titrated to 5 × 10^3^ (low dose), 5 × 10^4^ (mid dose) and 5 × 10^5^ CFU (high dose) and administered intranasally (i.n.) to C57BL/6 (B6) mice under anaesthesia. At 1, 7, or 14 days p.i. lungs were harvested for cytometry. **(B)** Confocal microscopy z-sections of a lung macrophage sorted by fluorescence-activated cell sorting as live, tdTomato+ 7 days post-infection. F4/80 staining (blue) shown with intracellular BCG tdTomato (red); slices taken at 20 nm steps. **(C)** B6 mice were infected i.n. with 5 × 10^5^ CFU BCG tdTomato, and lung homogenates plated on 7H11 agar ± HygB. Medians shown for *n* = 4–5 mice per group, data represent 2 independent experiments. **(D)** Representative plots showing tdTomato+ events among live, large (FSC^int/high^) or granular (SSC^int/high^) events 1–14 days following high dose (5 × 10^5^ CFU) i.n. infection with BCG tdTomato. **(E)** Representative plots showing cytometric gating strategy to subset lung phagocyte populations among singlet, live, large/granular events. Gates were set using unstained and fluorescence-minus-one (FMO) controls. Subset surface marker definitions are shown in Supplementary Table 3, subsets with lymphoid morphology (FSC^low^SSC^low^) were excluded from analysis. Int, intermediate.

To detect phagocytes participating in the early immune response to infection, mice were infected intranasally (i.n.) with tdTomato-expressing BCG (BCG tdTomato) and culled 1, 7, or 14 days post-infection (p.i.; Figure 1A). Confocal microscopy confirmed that phagocytes contained cytoplasmic bacilli (Figure 1B).

To test whether mycobacteria maintained the tdTomato plasmid *in vivo*, lung homogenates from infected mice were plated on agar with or without selective hygromycin (HygB). There was no difference in number of culturable colonies up to 14 days p.i., indicating that bacteria maintained the tdTomato plasmid at the times lungs were harvested in this study (Figure 1C). BCG tdTomato was detected by flow cytometry in live, FSC^int-high^SSC^int-high^ phagocytes at 1, 7, and 14 days p.i. (Figure 1D). Notably, the side-scatter profile of tdTomato^pos^ phagocytes was low on the first day p.i., and higher at 7 and 14 days p.i. Live lung phagocytes were classified using canonical lineage markers for lung-resident and peripheral myeloid cells using flow cytometry (Figure 1E and Supplementary Figure 1B and Supplementary Tables 1–3).

### Mycobacterial Infection Elicits Rapid Dose-Dependent Recruitment of Gr1^int^/Gr1^high^ Granulocytes

**FIGURE 2:**
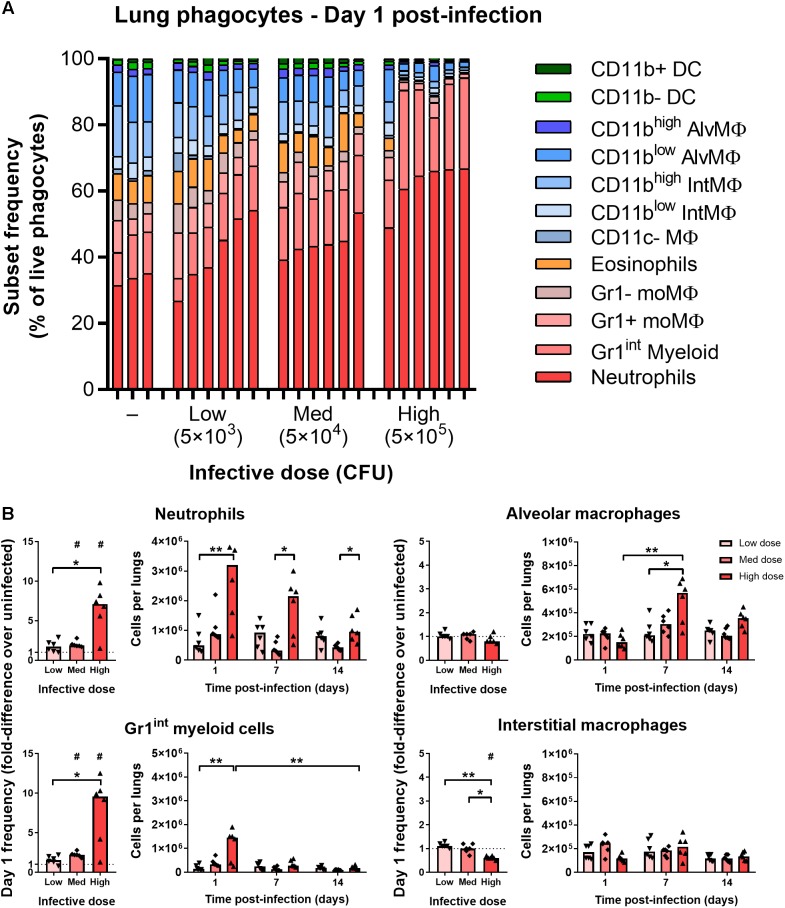
Mycobacterial infection elicits rapid dose-dependent recruitment of Gr1^int^/Gr1^high^ granulocytes. **(A)** Frequency of lung phagocyte subsets from naïve and BCG tdTomato-infected mice 1 day post-infection measured by flow cytometry and represented as parts-of-whole within the lung phagocyte compartment. Each bar represents one mouse, *n* = 3–6 per group. **(B)** Changes in phagocyte subset frequencies in the lungs of BCG-infected mice at 1 day post-infection, presented as fold-differences over the median value of uninfected mice and numbers of each subset recovered. Medians shown for *n* = 6 mice per group, data represent two independent experiments. Wilcoxon signed rank test for differences due to infection: ^#^*p* = 0.031. Kruskal–Wallis adjusted for multiple comparisons with Dunn’s post-test for differences by dose and time: ^∗^*p* ≤ 0.05, ^∗∗^*p* ≤ 0.01. MΦ, macrophage; AlvMΦ, alveolar macrophage; IntMΦ, interstitial macrophage; moMΦ, monocyte macrophage; Med, medium.

At 1 day after infection, mice that received low (5 × 10^3^ CFU) and medium (5 × 10^4^ CFU) doses of i.n. BCG tdTomato had a greater median frequency of Gr1^high^CD11b^high^ neutrophils in the lungs, by 1.7 and 2 times, respectively, than uninfected mice (Figure 2A,B). F4/80–CD11c–Gr1^int^CD11b+ phagocytes (referred to as “Gr1^int^ myeloid” cells) followed similar dose-response kinetics as neutrophils, increasing in median frequency by 1.6 and 2.2 times in mice given low and medium infective doses compared to uninfected mice (Figure 2B). In mice receiving the highest dose of BCG (5 × 10^5^ CFU), the frequency of neutrophils and Gr1^int^ myeloid cells increased by 7.5- and 9.5-fold – a significantly greater change than that induced by lower infective doses (Figure 2A,B). Of note, tdTomato^pos^ neutrophils (Gr1^high^CD11b^high^) were SSC^int^ on day 1 p.i., in contrast with tdTomato^neg^ neutrophils, which remained SSC^high^ (Supplementary Figure 1A). In contrast to uninfected mice and BCG-infected mice at later timepoints, when Gr1^int^ myeloid cells were Ly6C^high^Ly6G–, on day 1 p.i. these cells were Ly6C^int^Ly6G+ (indicating granulocyte differentiation), though entirely SSC^int^ (Supplementary Figure 1A).

By 7–14 days p.i., the number of neutrophils and Gr1^int^ myeloid cells was similar to uninfected mice (Figure 2B and Supplementary Figure 2A). Most of the mice that had received the highest infective dose maintained greater numbers of neutrophils at day 7 p.i. (Figure 2B and Supplementary Figure 2A). Dendritic cells (F4/80–CD11c^high^) did not increase in frequency on day 1 post-infection, though did appear to be recruited to the lungs with dose-dependent kinetics by 14 days post-infection (Supplementary Figure 2B). Taken together, and consistent with previous reports of the early phagocyte response to intradermal (Abadie et al., 2005) and intranasal BCG (Zhang et al., 2009), infection caused rapid recruitment of granulocytes to the lungs, in proportion to the quantity of infecting mycobacteria.

### Most BCG-Associated Phagocytes Are Gr1^int^/Gr1^high^ 1 Day Post-infection, Followed by Alveolar Macrophages 7–14 Days Post-infection

**FIGURE 3:**
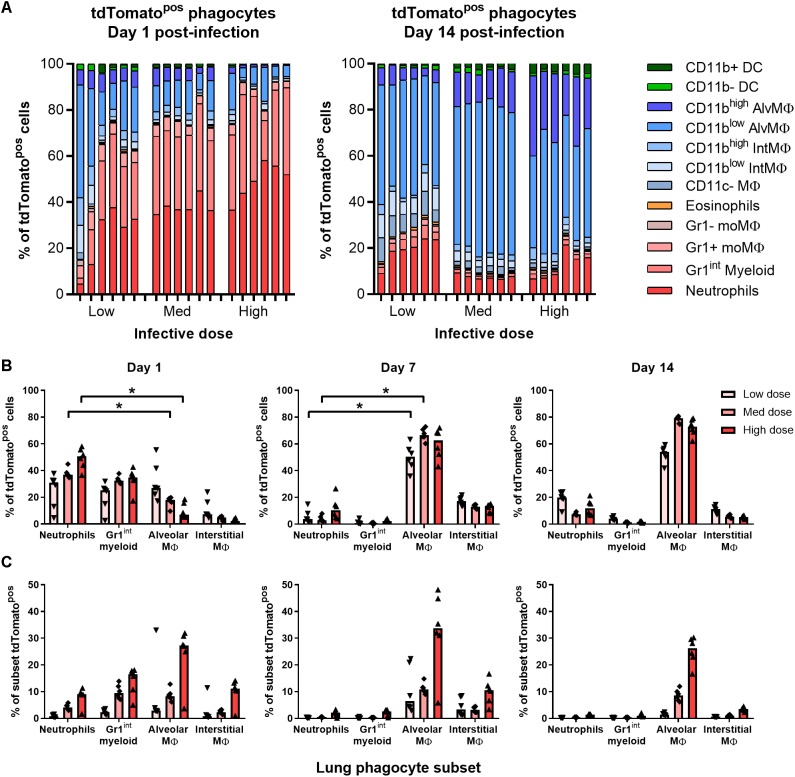
Most BCG-associated phagocytes are Gr1^int^/Gr1^high^ 1 day post-infection, followed by alveolar macrophages 7–14 days post-infection. Contribution of each phagocyte subset to the tdTomato+ compartment in the lungs of BCG-infected mice, shown as **(A)** parts-of-whole of live, tdTomato+ phagocytes, each bar representing one mouse, and **(B)** per subset for BCG-associated phagocytes during the first 2 weeks post-infection. **(C)** Frequency of BCG infection (tdTomato^pos^) per subset. Medians shown from *n* = 6 mice per group, data represent two independent experiments. Kruskal–Wallis adjusted for multiple comparisons with Dunn’s post-test for differences by dose and time: ^∗^*p* ≤ 0.05.

At all infective doses given i.n. (5 × 10^3^–5 × 10^5^ CFU), most tdTomato^pos^ phagocytes recovered from the lungs of mice 1 day p.i. were granulocytes (Figure 3A,B). At day 1 p.i., Gr1^high^ neutrophils and Gr1^int^ myeloid cells comprised 55% of infected cells in mice given the lowest infective dose of BCG tdTomato, and 70% to 85% of cells after medium and high dose infection, respectively (Figure 3B). A smaller portion of tdTomato^pos^ cells were alveolar (CD11c^high^), interstitial (CD11c^int^) and Gr1+ macrophages.

By days 7 and 14 p.i., alveolar and interstitial macrophages were the predominant infected cells (75–90% of tdTomato^pos^ in combination; Figure 3A,B and Supplementary Figure 3A) at all infective doses. Notably, neutrophils still comprised 7–20% of infected phagocytes by 14 days p.i. (Figure 3B). None of the phagocyte subsets associated with BCG had more than 50% of the cell population infected, even at the highest dose administered. At 1 day p.i., mice infected with the lowest infective dose had fewer than 5% of neutrophils, Gr1^int^ myeloid cells or any macrophage subset infected with BCG (Figure 3C). Surprisingly, CD11c+CD103+ DCs did not increase in number or appear to take up mycobacteria after infection (Supplementary Figures 3A,B).

### Prior Immunization With BCG Enhanced Abundance and Recruitment of Lung Macrophages

**FIGURE 4:**
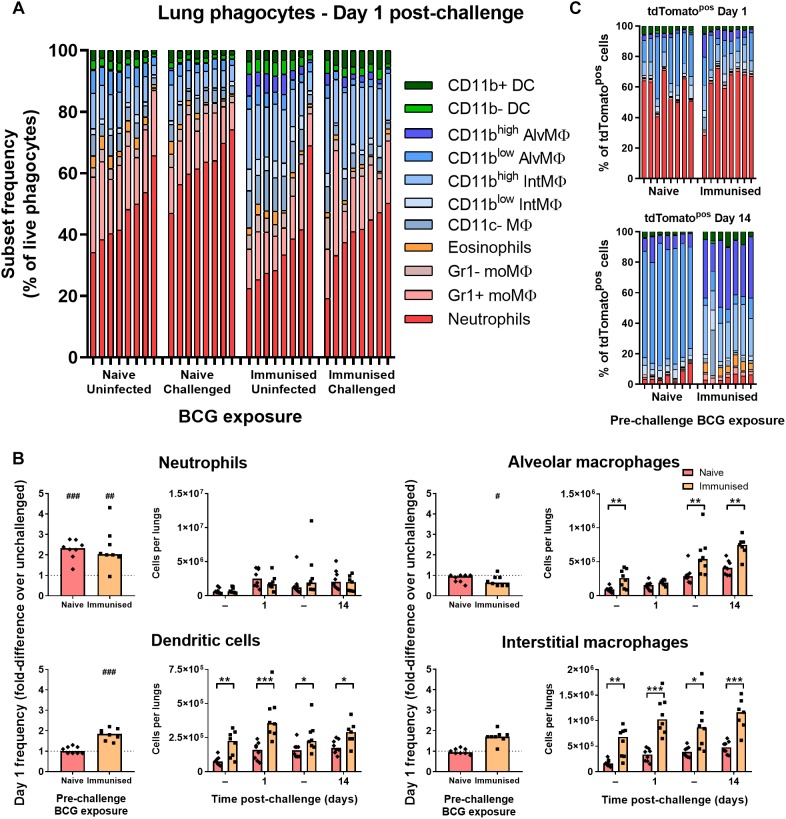
Prior immunization with BCG enhanced abundance and recruitment of lung macrophages. **(A)** Frequency of lung phagocyte subsets from uninfected and BCG tdTomato-infected mice 1 day after i.n. administration of 5 × 10^4^ CFU BCG tdTomato (challenged) or vehicle control (uninfected), represented as parts-of-whole within the lung phagocyte compartment. Groups of mice received either 10^5^ CFU BCG Pasteur i.n. (immunized) or vehicle control (naïve) 9 weeks prior to challenge. Each bar represents one mouse of *n* = 8 per group. Data represent two independent experiments. **(B)** Changes in phagocyte subset abundance in the lungs of BCG-infected mice 1 day post-infection (p.i.) presented as fold-differences in frequency over the median value of uninfected mice (dotted line), and absolute numbers of cells recovered 1 or 14 days p.i. from mice given 5 × 10^4^ CFU BCG tdTomato (Challenged) or vehicle control (Tween-PBS; Uninfected, –) i.n. Medians shown for *n* = 7–8 mice per group, data represent two independent experiments. Wilcoxon signed rank test for differences due to infection: ^#^*p* = 0.031, ^##^*p* = 0.01563, ^###^*p* = 0.007813. Mann–Whitney *U*-test for differences between naïve and immunized groups: ^∗^*p* ≤ 0.05, ^∗∗^*p* ≤ 0.01, ^∗∗∗^*p* ≤ 0.001. **(C)** Contribution of each phagocyte subset to the tdTomato+ compartment in the lungs of BCG-infected mice, shown as parts-of-whole. Each bar represents one mouse from *n* = 7–8 mice per group. Data represent two independent experiments.

To test whether the early phagocyte response is modulated in immunized B6 mice, groups were immunized with 10^5^ CFU BCG Pasteur i.n. 9 weeks prior to challenge infection with BCG tdTomato. In BCG-immunized mice, the frequency of lung neutrophils (including Ly6G+ Gr1^int^ myeloid cells here) among phagocytes and all live cells (Supplementary Figure 4A) was approximately half that of naïve mice (Figure 4A and Supplementary Figure 4B). Although the absolute frequency of neutrophils was also significantly lower in immunized mice compared to unimmunized mice 1 day p.i. (Supplementary Figure 4B), neither recruitment (fold-difference in frequency over unchallenged controls), nor absolute number of neutrophils post-challenge were significantly altered by prior BCG immunization (Figure 4B).

The reduced frequency of neutrophils in immunized mice was attributable instead to the presence of greater numbers of dendritic cells, alveolar, interstitial (Figure 4B) and CD11c– macrophages (data not shown) in the lungs prior to and following challenge infection. Unlike unimmunized mice, the frequency of interstitial macrophages and dendritic cells increased further in immunized mice at 1 day p.i., suggesting prior BCG exposure enhanced recruitment of these cells to the lungs early after challenge (Figure 4B). The frequency of neutrophils among tdTomato^pos^ phagocytes was not reduced in immunized mice; neutrophils comprised ∼60% of infected cells 1 day p.i. in both groups (Figure 4C). Although macrophage subsets again became the majority of infected cells by 14 days p.i. in both challenged groups, tdTomato^pos^ cells were more frequently CD11b^high^ (Figure 4C). While CD11c^high^ alveolar macrophages comprised nearly all infected cells in previously BCG-naïve mice 14 days after challenge, the infected macrophage compartment in immunized mice contained a sizable portion of CD11c^int^ interstitial macrophages as well (Figure 4C and Supplementary Figure 4C).

### Immunization With BCG Enhances CD11b Expression on Lung Macrophages

**FIGURE 5:**
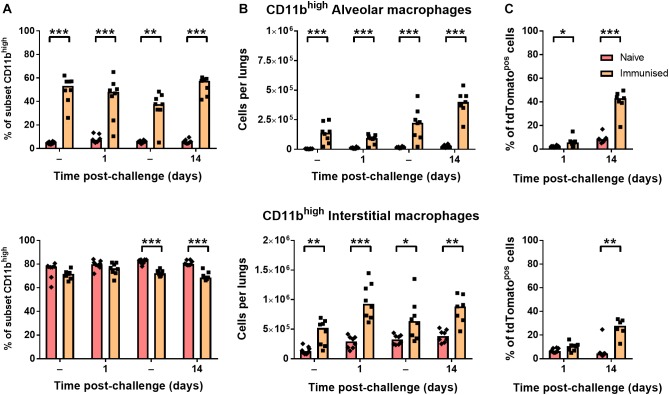
Immunization with BCG enhanced resident populations of CD11b^high^ macrophages. Differences in **(A)** frequency and **(B)** number of CD11b^high^ alveolar (CD11c^high^; top) and interstitial (CD11c^int^; bottom) macrophages, and **(C)** their proportion within the tdTomato+ compartment at 1 or 14 days post-infection in mice given BCG tdTomato or vehicle control (–) i.n. Medians shown for *n* = 7–8 mice per group, data represent two independent experiments, *n* = 7–8. Mann–Whitney *U*-test for differences between naïve and immunized groups: ^∗^*p* ≤ 0.05, ^∗∗^*p* ≤ 0.01, ^∗∗∗^*p* ≤ 0.001.

CD11b-expressing alveolar macrophages were significantly greater in both frequency (Figure 5A) and number (Figure 5B) in immunized mice before and after challenge infection (∼50% CD11b^high^) compared to naïve mice (<10%). Although interstitial macrophages were near-uniformly CD11b^high^ irrespective of BCG exposure (Figure 5A), the number of CD11b^high^ interstitial macrophages was also significantly greater in immunized mice (Figure 5B). As a result, a significantly greater proportion of tdTomato^pos^ phagocytes were CD11b^high^ macrophages after challenge infection, particularly by 14 days p.i. where CD11b^high^ AMΦ constituted ∼40% of infected cells (Figure 5C). Together, these data indicate that while neutrophil accumulation was unchanged in i.n.-immunized B6 mice, prior BCG exposure did lead to alterations to the abundance and recruitment of macrophage and DC subsets. Most notably, the frequency of CD11b^high^ macrophages among tdTomato^pos^ phagocytes was 2.5–4.5 times greater in immunized mice at 1 and 14 days p.i., respectively.

## Discussion

In this study, we characterized the early phagocytic events in the lungs of mice after mycobacterial infection. We found that contrary to common dogma, granulocytes, rather than alveolar macrophages, were most commonly associated with mycobacteria on the first day after infection. In line with studies at later timepoints after infection of rabbits and mice (Cunningham et al., 1925; Wolf et al., 2007), we found that macrophages had become the main infected phagocytes by 7 days p.i. In addition, we showed that mucosal BCG immunization modulated the early phagocytic response in the lung to a subsequent BCG challenge, by increasing the number of CD11b^high^ macrophages compared to unimmunized and challenged mice. The observed rapid granulocyte response in the lung after mycobacterial exposure begs that greater consideration be given to the earliest innate immune response, particularly the variety of niches the bacteria may be exposed to during the initial stage of infection. These events may influence the effectiveness and outcome of the ensuing innate and adaptive responses.

We found that the magnitude of granulocyte infiltration depended on the infective dose, however, even after infection with 5 × 10^3^ CFU of BCG tdTomato, granulocytes were the predominant BCG-associated phagocytes on the first day after infection. At this low dose fewer than 10% of AMΦ were tdTomato^pos^, suggesting the abundance of infected granulocytes was not merely due to surplus bacilli saturating resident macrophages. It would be valuable to determine how consistently granulocyte infiltration features after low-dose aerosol infection – particularly with virulent Mtb, and in mouse strains with differing susceptibility to infection and differing lung lesion structures.

C57BL/6 mice are considered genetically resistant to mycobacteria, and the lung infiltrate described here does not precede the development of granulomatous disease seen in humans and some other animal models (North and Jung, 2004). Still, following aerosol infection with Mtb, B6 mice eventually succumb to disease when control of bacterial replication is lost (Almeida et al., 2017). Although B6 mice represent only one example of a genotypic response to mycobacterial infection, their mononuclear phagocyte system is well-characterized and provides a degree of useful comparison with humans (Serbina et al., 2008). Importantly, B6 mice have been used to generate key paradigms of TB infection (Orme et al., 2001), and provide the most extensively studied background against which to model and contrast the events of early infection. Nonetheless, it will be essential to investigate early events in the lungs of strains of mice considered susceptible to Mtb infection.

Human neutrophils isolated from the blood of Mtb case-contacts produce anti-mycobacterial peptides, and are associated with a lower risk of infection (IGRA conversion) (Martineau et al., 2007). Though potentially protective, neutrophil degranulation can contribute to TB disease (Palanisamy et al., 2011; Mattila et al., 2015). Blood neutrophil count at the time of clinical TB diagnosis is associated with poor short-term prognosis (Barnes et al., 1988), and in highly susceptible mice and non-human primates, persistent neutrophil infiltration is associated with necrotic lesions (Gopal et al., 2013; Yeremeev et al., 2015; Smith et al., 2016).

Similarly, in TB-infected mice, a greater neutrophil infiltration and higher concentration of neutrophil-attractant chemokines in the lungs correlate with increased lung disease (Niazi et al., 2015). In mouse strains susceptible to necrosis and mortality after Mtb infection, neutrophils accumulated in the lungs by 3 weeks p.i. and did not contract in subsequent weeks, as they did in resistant strains (Smith et al., 2016). In humans, severity of TB disease has recently been found to correlate more strongly with neutrophil haematopoiesis, than with blood lymphocyte abundance or functionality (Panteleev et al., 2017). This broad range of evidence from animals and humans highlights a link between lung neutrophils and severity of disease after mycobacterial infection.

Although antibody-mediated depletion of neutrophils before infection did not affect control of i.n. BCG in resistant B6 mice by 3 days p.i. (Zhang et al., 2009), in hyper-susceptible I/St mice neutrophil depletion 2 and 6 days after infection with Mtb reduced tissue damage, mortality and number of lung bacteria at 4 weeks p.i., but not in resistant B6 mice (Yeremeev et al., 2015). These data suggest that while neutrophils may be unnecessary for early restriction of mycobacterial replication, their influence on the outcome of infection differs between resistant and susceptible hosts. In particular, they implicate persistent neutrophil infiltration after early infection as a contributing factor to TB disease.

In cattle infected experimentally and naturally with virulent *M. bovis*, scattered neutrophils can be found in low numbers at all stages of lesion development (Wangoo et al., 2005), though are most abundant at the least progressive stages of granuloma formation (I–II) (Menin et al., 2013). By days 7–15 post-infection when lesions begin to develop in the lungs, accumulation of epithelioid macrophages is more pronounced than that of neutrophils (Palmer et al., 2007). However, neutrophils associated with acid-fast bacilli can be found in the lungs as early as 7 days post-infection, along with macrophages containing neutrophilic debris (Cassidy et al., 1998). These data from early bovine infection and disease suggest uptake of bacilli by macrophages may occur secondary to initial uptake by and subsequent turnover of neutrophils in the natural host for *M. bovis*.

While our data demonstrate major involvement of granulocytes early after infection, it is clear that macrophages presume the role of primary hosts to the bacilli soon after infection. In our study, alveolar macrophages were typically CD11b–/^low^ up to 14 days p.i., however, this differed in mice previously exposed to i.n. BCG. In these immunized mice, half of all CD11c^high^ macrophages were consistently CD11b^high^, and CD11c^high^ macrophages were present in significantly greater number in immunized mice compared to unimmunized controls. Whether this CD11c^high^CD11b^high^population has enhanced anti-mycobacterial capacity and contributes to the protective effect of BCG immunization, or is simply an eventual consequence of intranasal mycobacterial exposure (either by upregulation of the integrin or recruitment from parenchymal macrophages) requires further investigation.

Alveolar macrophages, identified as F4/80+CD11c^high^ in mice, are generally CD11b–/^low^ (Gonzalez-Juarrero et al., 2003; Srivastava et al., 2014). AMΦ can become CD11b+/^high^ under the influence of LPS (Duan et al., 2012), IL-1α (Huaux et al., 2015), and after replenishment from peripheral mononuclear cells (Kirby et al., 2006), and in these contexts are associated with enhanced inflammatory and phagocytic capacity. Determining whether the regulatory and functional nature of these cells is distinct from their CD11b–/^low^ counterparts will contribute to our understanding the role they play in defense against mycobacterial infection. In particular, the capacity for these and other infected phagocyte subsets to produce effector molecules (including reactive oxygen and nitrogen species) early after infection is of great interest.

Wolf et al. identified CD11c^low^CD11b^high^ recruited macrophages as a low frequency subset in the lungs of uninfected B6 mice which increased in frequency from 14 days p.i., and constituted a major chronically infected subset after aerosol Mtb challenge (Wolf et al., 2007). Our data, from earlier in infection, showed that interstitial (CD11c^int^) macrophages were almost uniformly CD11b^high^, and increased in number concurrent with the expansion of alveolar (CD11c^high^) macrophages in immunized mice. If the expansion of either compartment draws from circulating leukocytes after immunization, or by enhanced recruitment during early infection, the fact that CD11b^high^ macrophages constituted a greater proportion of infected cells in immunized mice early after infection would suggest an opportunity for training these cells or their precursors.

Norris and Ernst found that CD11c^low^CD11b+ recruited macrophages were over twice as frequent in the lungs of Mtb-infected mice 16 weeks p.i., and that at least a fraction of these cells derived from transferred Ly6C^high^ precursors (Norris and Ernst, 2018). Further, they showed that EdU-labeled recruited macrophages peaked and contracted within 7 days of EdU pulse, suggesting a relatively high rate of turnover in the lungs. Even during the chronic phase of infection, up to 50% of infected neutrophils and monocytes had recently-proliferated (EdU+) (Norris and Ernst, 2018). These data suggest that mycobacteria elicit continuous trafficking of short-lived phagocytes to the lungs, which would explain the increased abundance and altered surface marker expression of macrophages we found in the lungs of immunized mice. Importantly, the opportunity for replenishment of lung phagocytes provides opportunities for training and host-directed therapy. It will be critical to determine whether similar expansion can be elicited by parenteral (intradermal or subcutaneous) immunization, and how essential alterations to macrophage recruitment are to protection afforded by BCG vaccination.

Our study of the very early events after mycobacterial infection reveals a rapid and dynamic lung phagocyte response to slow-growing mycobacteria, implicating early-infiltrating granulocytes as a major subset taking up bacilli during the earliest stage of infection. Notably, many more Gr1^int^/Gr1^high^ phagocytes took up mycobacteria on the first day of infection than did AMΦ, providing contrast to the prevailing dogma that AMΦ are the principle determinants of the outcome of early mycobacterial infection. We also show that immunization with BCG alters lung-resident macrophage populations. These findings provide greater understanding of the early innate immune response to mycobacteria, and lend support to the development of methods to train innate immunity against infection.

## Data Availability

The datasets generated for this study are available on request to the corresponding author.

## Author Contributions

SW and JD developed BCG expressing tdTomato. BR, SS, KM, and JK designed and performed the experiments. BR, KM, and JK interpreted the data. BR and JK wrote the manuscript.

## Conflict of Interest Statement

The authors declare that the research was conducted in the absence of any commercial or financial relationships that could be construed as a potential conflict of interest.
