# Supplementary figures and images for: Gr1int/high Cells Dominate the Early Phagocyte Response to Mycobacterial Lung Infection in Mice
Source: Front Microbiol. 2019 Mar 8;10:402. doi: 10.3389/fmicb.2019.00402 (PMC6418015; doi:10.3389/fmicb.2019.00402)

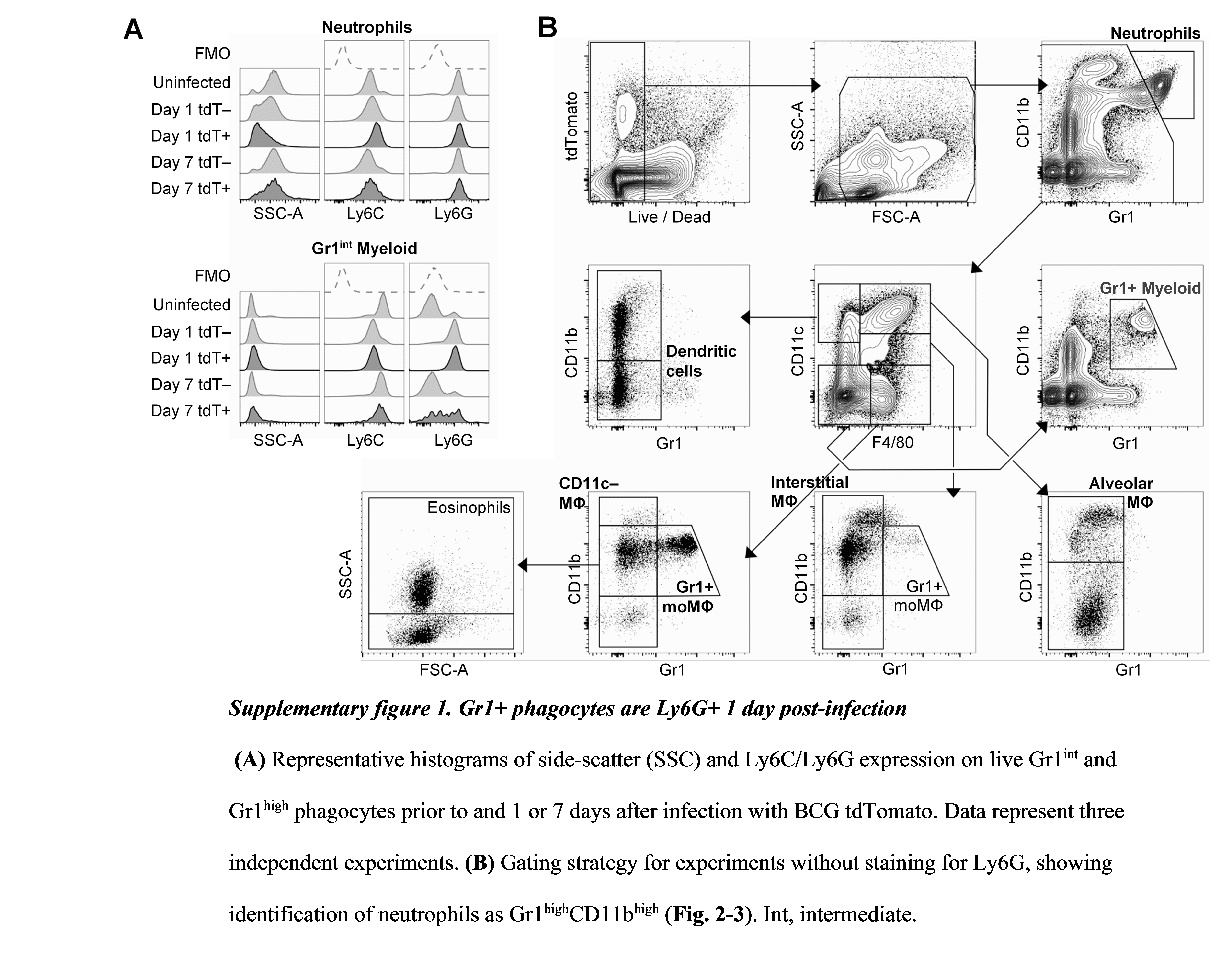

Supplement: Supplementary file 1 [file Image_1.TIF]

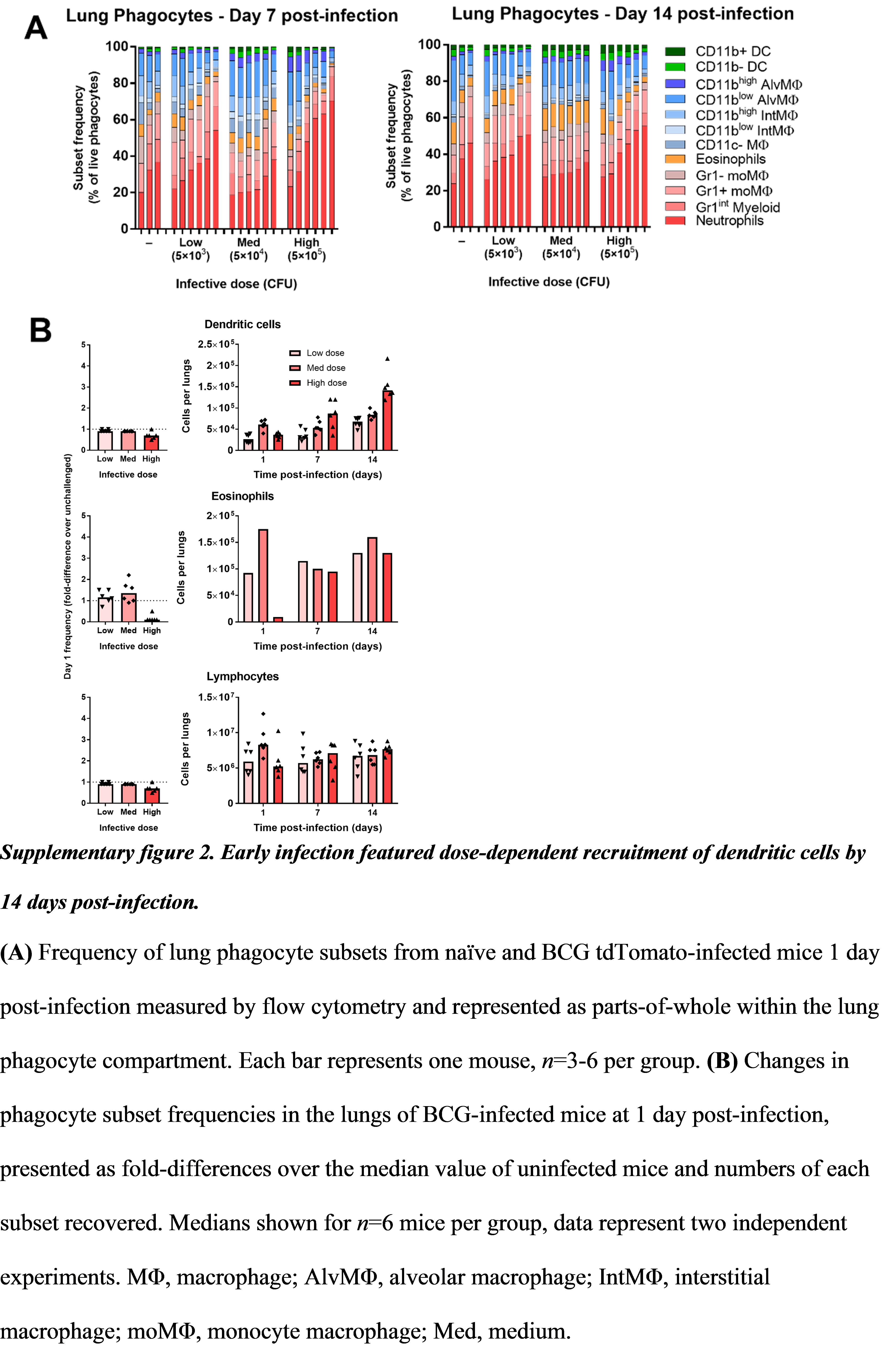

Supplement: Supplementary file 2 [file Image_2.TIF]

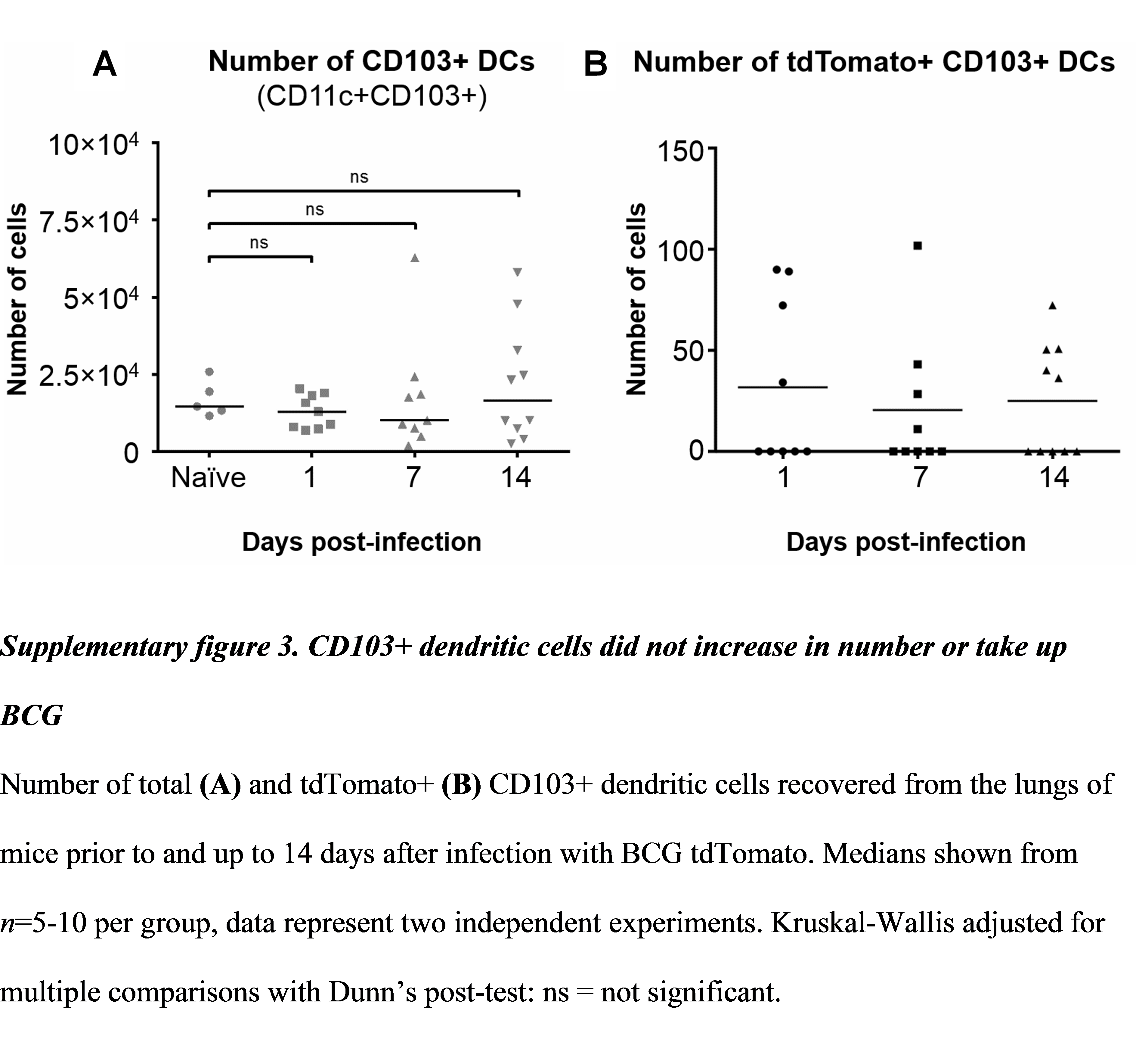

Supplement: Supplementary file 3 [file Image_3.TIF]

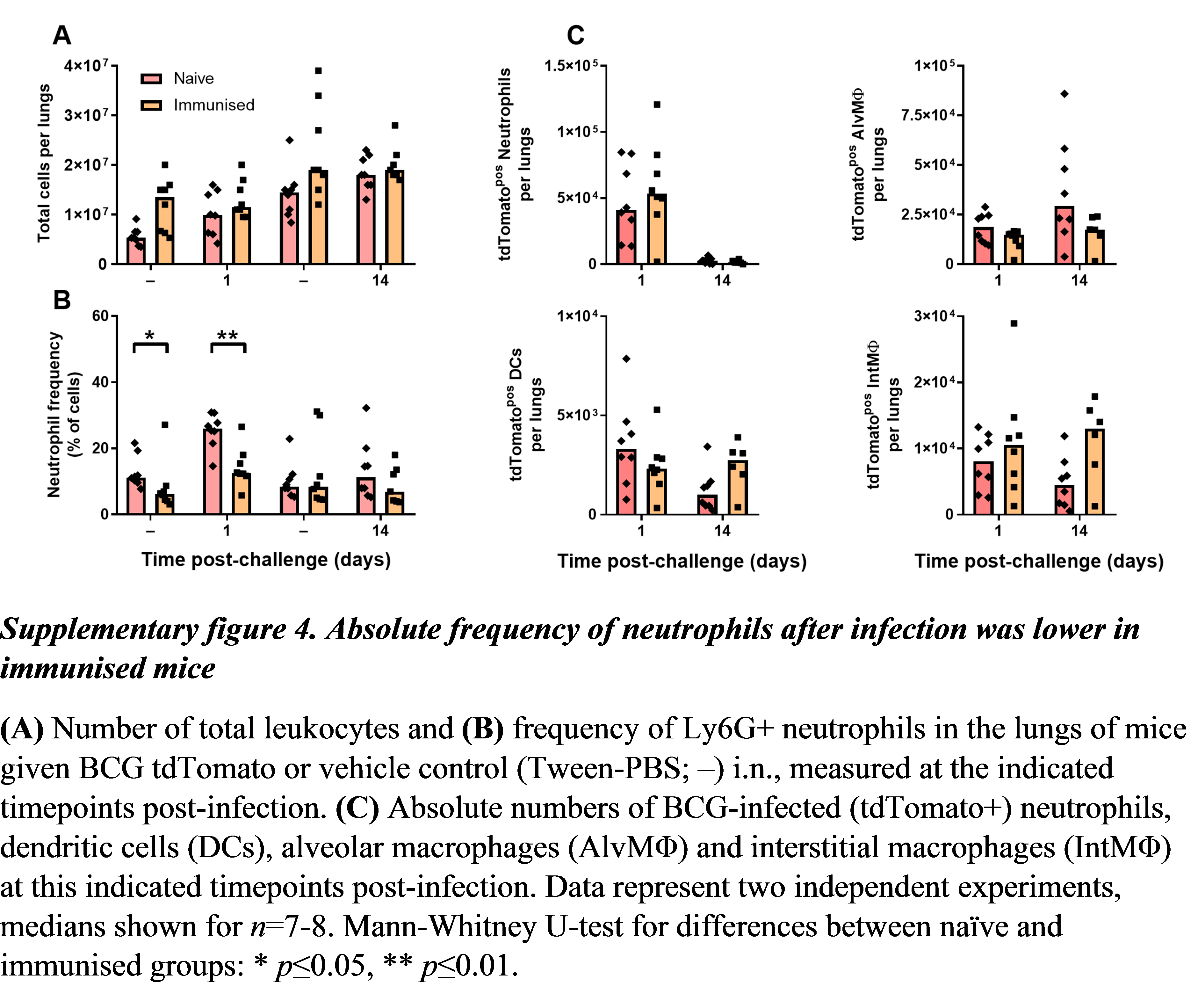

Supplement: Supplementary file 4 [file Image_4.TIF]
