# Supplementary material for: Gr1int/high Cells Dominate the Early Phagocyte Response to Mycobacterial Lung Infection in Mice
Source: Front Microbiol. 2019 Mar 8;10:402. doi: 10.3389/fmicb.2019.00402 (PMC6418015; doi:10.3389/fmicb.2019.00402)
Supplement: Supplementary file 5 [file Table_1.DOCX]

# Supplementary table 1. Lung phagocyte cytometry panel

| **Target** | **Conjugate** | **Species / Isotype** | **Clone** | **Manufacturer** | **Cat. #** | **RRID** | **Machine citation** |
| --- | --- | --- | --- | --- | --- | --- | --- |
| CD16/32 | - | Rat anti-mouse IgG2b, κ | 2.4G2 | In-house | - | - | - |
| Viability | FVS700 | Amine-reactive | - | BD Pharmingen | 564997 | - | - |
| Gr1 | PerCP-Cy5.5 | Rat anti-mouse IgG2b, κ | RB6-8C5 | BD Pharmingen | 552093 | AB_394334 | (BD Biosciences Cat# 552093, RRID:AB_394334) |
| Ly6C | BV421 | Rat anti-mouse IgM, κ | AL-21 | BD Pharmingen | 562727 | AB_2737748 | (BD Biosciences Cat# 562727, RRID:AB_2737748) |
| Ly6G | BV605 | Rat anti-mouse IgG2a, κ | 1A8 | BD Pharmingen | 563005 | AB_2737946 | (BD Biosciences Cat# 563005, RRID:AB_2737946) |
| CD11b | APC-Cy7 | Rat anti-mouse IgG2b, κ | M1/70 | Biolegend | 101226 | AB_830642 | (BioLegend Cat# 101226, RRID:AB_830642) |
| CD11c | PE-Cy7 | Hamster anti-mouse IgG2 | N418 | Biolegend | 117317 | AB_493569 | (BioLegend Cat# 117317, RRID:AB_493569) |
| F4/80 | APC | Rat anti-mouse IgG2a, κ | BM8 | Biolegend | 123116 | AB_893481 | (BioLegend Cat# 123116, RRID:AB_893481) |
| CD103 | APC | Hamster anti-mouse IgG2 | 2E7 | Biolegend | 121413 | AB_1227503 | (BioLegend Cat# 121413, RRID:AB_1227503) |
