# Supplementary material for: Gr1int/high Cells Dominate the Early Phagocyte Response to Mycobacterial Lung Infection in Mice
Source: Front Microbiol. 2019 Mar 8;10:402. doi: 10.3389/fmicb.2019.00402 (PMC6418015; doi:10.3389/fmicb.2019.00402)
Supplement: Supplementary file 6 [file Table_2.DOCX]

# Supplementary table 2. LSR Fortessa optical configuration

| **Laser** | **LP λ** | **BP λ** | **Antigen** | **Fluorophore** |
| --- | --- | --- | --- | --- |
|  |  |  |  |  |
| 405 |  | 450/50 | *Ly6C* | *BV421* |
|  | 505 | 525/50 |  |  |
|  | 690 | 710/50 |  |  |
|  | 600 | 610/20 | *Ly6G* | *BV605* |
|  |  |  |  |  |
| 488 | 505 | 530/30 | Empty | Autofluorescence |
|  | 685 | 710/50 | Gr1 | PerCP-Cy5.5 |
|  |  |  |  |  |
| 561 |  | 586/15 | BCG | tdTomato |
|  | 600 | 610/20 |  |  |
|  | 635 | 670/30 |  |  |
|  | 685 | 710/50 |  |  |
|  | 750 | 780/60 | CD11c | PE-Cy7 |
|  |  |  |  |  |
| 640 |  | 670/14 | F4/80 | APC |
|  | 690 | 730/45 | Live/dead | FVS700 |
|  | 750 | 780/60 | CD11b | APC-Cy7 |
