# Supplementary material for: Gr1int/high Cells Dominate the Early Phagocyte Response to Mycobacterial Lung Infection in Mice
Source: Front Microbiol. 2019 Mar 8;10:402. doi: 10.3389/fmicb.2019.00402 (PMC6418015; doi:10.3389/fmicb.2019.00402)
Supplement: Supplementary file 7 [file Table_3.DOCX]

# Supplementary table 3. Phagocyte subset definitions

Neutrophils were identified either by expression of Ly6G (**Fig. 4**) or by pre-gating on Gr1^high^CD11b^high^ as shown in **Fig. S1B** (**Fig. 2-3**). F4/80+ events were classified as macrophages, subsetted first as CD11c^high^ (alveolar MΦ), CD11c^int^ (interstitial MΦ), or CD11c–, then further by expression of CD11b. Dendritic cells were identified as F4/80–CD11c^high^. Monocytic origin was determined by expression of Gr1/Ly6C and/or by progressively decreasing Ly6C expression in Gr1–CD11b+ cells. Int, intermediate.

|  | Neutrophils | Gr1^int^ Myeloid | Gr1+ monocyte MΦ | Gr1– monocyte MΦ | Eosinophils | CD11c– MΦ | Interstitial MΦ | Alveolar MΦ | CD11b– DC | CD11b+ DC |
| --- | --- | --- | --- | --- | --- | --- | --- | --- | --- | --- |
| FSC | Int | Int | Int | Int | Int | Int / High | Int | High | Int | High |
| SSC | Int / + | Int / + | Int | Int | High | Low / High | Int | High | Int | High |
| Gr1 | High | Int | Int | – | – | – | – | – | – | – |
| Ly6G | + | – / + | – | – | – / Low | – | – | – | – | – |
| Ly6C | Int | Int / High | High | – / Low | – / Low | – | – | – | – | – |
| F4/80 | – | – | + | + | + | + | + | + | – | – |
| CD11c | – | – | – / + | – / + | – | – | Int | High | High | High |
| CD11b | High | + | + | + | + | Low / High | Low / High | Low / High | – | + / High |
