# Supplementary material for: Gr1int/high Cells Dominate the Early Phagocyte Response to Mycobacterial Lung Infection in Mice
Source: Front Microbiol. 2019 Mar 8;10:402. doi: 10.3389/fmicb.2019.00402 (PMC6418015; doi:10.3389/fmicb.2019.00402)
Supplement: Supplementary file 8 [file Table_4.DOCX]

# Supplementary table 4. Specific pathogen-free status for mice used in this study

Sentinel animals were tested twice annually for the specified pathogens. Methods as indicated: PCR, polymerase chain-reaction; EIA/IFA, enzyme-linked immunosorbent assay / immunofluorescence assay.

| **Species** | **Strain** |  |
| --- | --- | --- |
| Mouse | C57BL/6 |  |
|  |  |  |
| **Bacteria and Fungi** | **Pos/tested** | **Method** |
| *Helicobacter* spp. | 0/1 | PCR |
|  |  |  |
| **Endoparasite** |  |  |
| Pinworm - *Aspiculuris tetraptera* | 0/1 | PCR |
| Pinworm - *Syphacia obvelata* | 0/1 | PCR |
|  |  |  |
| **Viruses** |  |  |
| Minute Virus of Mice | 0/2 | EIA/IFA |
| Mouse Hepatitis Virus | 0/2 | EIA/IFA |
| Mouse Norovirus | 0/2 | EIA/IFA |
| Mouse Parvovirus | 0/2 | EIA/IFA |
| Rotavirus | 0/2 | EIA/IFA |
| Theiler’s Encephalomyelitis virus | 0/2 | EIA/IFA |
